# Color inference in visual communication: the meaning of colors in recycling

**DOI:** 10.1186/s41235-018-0090-y

**Published:** 2018-02-21

**Authors:** Karen B. Schloss, Laurent Lessard, Charlotte S. Walmsley, Kathleen Foley

**Affiliations:** 1 Department of Psychology, University of Wisconsin–Madison, 1202 West Johnson St., Madison, WI 53706 USA; 2 Wisconsin Institute for Discovery, University of Wisconsin–Madison, 330 N Orchard St., Madison, WI 53715 USA; 3 Department of Electrical and Computer Engineering, University of Wisconsin–Madison, 1415 Engineering Dr., Madison, WI 53706 USA; 4 Termeer Center for Targeted Therapies, Massachusetts General Hospital Cancer Center, Boston, MA 02114 USA

**Keywords:** Color cognition, Information visualization, Visual reasoning

## Abstract

People interpret abstract meanings from colors, which makes color a useful perceptual feature for visual communication. This process is complicated, however, because there is seldom a one-to-one correspondence between colors and meanings. One color can be associated with many different concepts (one-to-many mapping) and many colors can be associated with the same concept (many-to-one mapping). We propose that to interpret color-coding systems, people perform assignment inference to determine how colors map onto concepts. We studied assignment inference in the domain of recycling. Participants saw images of colored but unlabeled bins and were asked to indicate which bins they would use to discard different kinds of recyclables and trash. In Experiment 1, we tested two hypotheses for how people perform assignment inference. The local assignment hypothesis predicts that people simply match objects with their most strongly associated color. The global assignment hypothesis predicts that people also account for the association strengths between all other objects and colors within the scope of the color-coding system. Participants discarded objects in bins that optimized the color-object associations of the entire set, which is consistent with the global assignment hypothesis. This sometimes resulted in discarding objects in bins whose colors were weakly associated with the object, even when there was a stronger associated option available. In Experiment 2, we tested different methods for encoding color-coding systems and found that people were better at assignment inference when color sets simultaneously maximized the association strength between assigned color-object parings while minimizing associations between unassigned pairings. Our study provides an approach for designing intuitive color-coding systems that facilitate communication through visual media such as graphs, maps, signs, and artifacts.

**Electronic supplementary material:**

The online version of this article (10.1186/s41235-018-0090-y) contains supplementary material, which is available to authorized users.

## Significance

This article examines how people interpret messages encoded in color-coding systems. Such systems are prevalent in a variety of domains: in hospitals, where syringe colors code for different classes of anesthetic drugs and wristband colors code for different kinds of patient risks; in animal shelters, where cage sign colors indicate how challenging the animal is to handle; and in recycling, where bin colors code for different kinds of objects to be discarded. Color is a useful visual feature for communicating because it can be observed quickly from a distance and it can signal a variety of messages, ranging from “approach this animal with caution,” to “put paper in this bin.” However, with no one-to-one mapping between colors and concepts, how can messages be most effectively and efficiently encoded in colors? We addressed this question by investigating how observers interpret color-coding in recycling. We found that people have expectations for how different colored bins signal different kinds of to-be-discarded objects (e.g. paper, glass, trash), based on their color-object associations and contextual cues from other colors in the set. They responded as though they were solving a global assignment problem, which optimizes the color-object associations of the entire set. By understanding the principles by which people map perceptual features onto abstract concepts, we can use those principles to make visual communication more effective and efficient.

## Background

People can interpret complex messages encoded in visual features. They know red splotches on a weather map signal impending storms, red traffic lights signal stop, and red milk cartons signal that the container holds whole milk. Given this ability, people use colors to communicate important and time-sensitive information. For example, a recent surgical protocol for separating conjoined twins used green and purple tape to signal which monitors and equipment were dedicated to each twin (Associated Press, 2017), presumably so they did not get mixed up during surgery.

Color is one of many visual features that can be used to communicate abstract information, with others including size, texture, orientation, and shape (Bertin, 1983; Ware, 2012). However, color is especially useful for signaling because it can be observed quickly from a distance and it provides meaningful information that is independent from spatial structure. In nature, changes in face color can signal changes in emotional state independent of facial features and changes in fruit color signal ripeness independent from changes in shape (Lafer-Sousa, Conway, & Kanwisher, 2016; Thorstenson, Elliot, Pazda, Perrett, & Xiao, 2017). In human-made artifacts, differences in font color can signal different meanings in signs and maps without affecting legibility of the text. People even make inferences about student ability and teacher competence based on the ink color used to provide feedback on essays (Richards & Fink, 2017). Most relevant to the present study, differences in surface colors can signal different kinds of recycling bins without interfering with the ability to insert objects into the bins.

**Figure 1:**
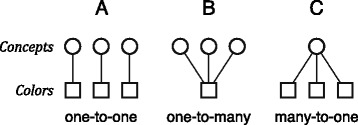
Mappings between colors (*squares*) and concepts (*circles*) that are **a** one-to-one, **b** one-to-many, and **c** many-to-one

Yet, interpreting colors is complicated because there is no one-to-one correspondence between colors and concepts (Fig. 1a) in nature or the human-made world (Elliot & Maier, 2012; Humphrey, 1976; Lin, Fortuna, Kulkarni, Stone, & Heer, 2013; Setlur & Stone, 2016). There are one-to-many mappings (Fig. 1b), in which the same color is associated with multiple concepts (e.g. red associated with ripe apples, strawberries, fire, the US Republican Party, and the University of Wisconsin–Madison) and many-to-one mappings (Fig. 1c), in which many colors are associated with the same concept (shades of reds, yellows, and greens associated with ripe apples) (Schloss & Heck, 2017). How, then, do observers interpret reliable and meaningful signals from colors?

We addressed this question by investigating how observers interpret colors in color-coding systems designed for visual communication. When people attempt to communicate through visual media (e.g. graphs, maps, signs, and artifacts), two distinct tasks emerge. There is an encoding task, in which designers^1^ select perceptual features to signify concepts for a design, and a decoding task, in which observers interpret how perceptual features map onto concepts in the design (Cleveland & McGill, 1984; Wood, 1968). Ideally, observers will be able to decode the same message that was encoded by the designer.

This decoding ability depends on the degree to which encodings match people’s predicted mappings, or expectations (Norman, 1988, 2013; Tversky, 2011; Tversky, Morrison, & Betrancourt, 2002; Zacks & Tversky, 1999). For example, observers are faster at interpreting bar graphs depicting fruit sales when the bar colors match the colors of the fruit they represent (e.g. banana – yellow, blueberry – blue) than when they mismatch (e.g. banana – orange, blueberry – green) (Lin et al., 2013). One might argue that if color-coding systems are clearly labeled, then interpreting those systems is trivial—you just look up the answer. However, Lin et al. (2013) demonstrated that there is a processing cost to interpreting color-coding systems (even with clear labels) if they do not correspond to people’s predictions for how colors should map onto concepts. The question is, what determines people’s predicted mappings for color-coding systems?

We approach this question by considering visual communication as a set of assignment problems. In optimization and operations research, assignment problems (also known as maximum-weight matching problems) are mathematical models that describe how to pair items from two different categories (Kuhn, 1955; Munkres, 1957). Examples include optimally assigning employees to jobs in a company, machines to tasks in a factory, and trucks to routes in a shipping network (Williams, 2013; Winston & Goldberg, 2004).

Here, we consider two types of assignment problems for generating and interpreting color-coding systems, which correspond to the encoding and decoding tasks described above: *encoding assignment problems* and *decoding assignment problems*. Although we focus on color-coding systems here, the principles can generalize to any coding system in which concepts map onto perceptual features.

### Encoding assignment problem

**Fig. 2:**
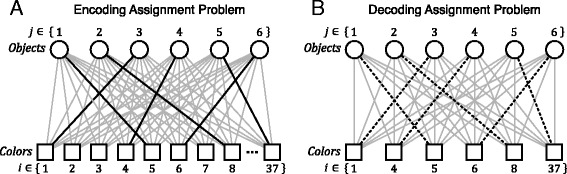
*Bipartite graphs* illustrating color-object assignments. **a** In encoding, the designer determines optimal assignments between colors and objects, given the merit scores. Here, there are more colors than objects. *Black edges* represent assigned color-object pairs and *gray edges* represent unassigned color-object pairs. **b** In decoding, observers try to infer how the designer encoded color-object assignments. *Dashed black edges* represent inferred assignments that match the encoded assignments

Designers can use encoding assignment problems to generate color-coding systems by determining optimal assignments between colors and concepts. Figure 2a illustrates an encoding assignment problem as a bipartite graph. There are 37 colors (denoted using index $i\in \left\{1,\dots, 37\right\}$) and six objects (denoted using index $j\in \left\{1,\dots, 6\right\}$). Here, and henceforth, we typically refer to "objects" instead of "concepts" because the focus of this paper is on color-coding systems for objects to be discarded in trash and recycling bins. The choice of numerical labels is arbitrary and only serves to simplify the explanation. Each potential pairing $\left(i,j\right)$ has a corresponding merit score, ${m}_{ij}$, which quantifies the desirability of pairing $i$ with $j$, computed using a merit function. Merit scores can be thought of as weights on each of the edges of the graph in Fig. 2a.

Solving an encoding assignment problem means to select a subset of the edges such that each object is assigned to exactly one color and the sum of merit scores along selected edges is maximized. In Fig. 2a, each assigned color-object pair is represented by a black edge and each unassigned color-object pair is represented by a gray edge. The optimal assignment will depend on the particular choice of merit scores.^2^

Lin et al. (2013) used this kind of approach to study how the association strength between colors and concepts influenced people’s ability to interpret color-coding systems in bar graphs. To encode the color-concept pairings for their test stimuli (e.g. graphs of fruit sales), they first obtained frequency distributions of colors in Google Image Search for a series of concepts (e.g. fruits) and then interpreted the resulting color-concept histograms as probability distributions. The authors then computed a merit function, called an affinity, by weighting each probability of a color occurring for a given concept by the inverse of the entropy of that color’s probability distribution across all concepts. This approach rewards strong color-object associations for the intended pairing while penalizing the associations of unintended pairings. Another way to achieve this qualitative property is to use the pointwise mutual information, another information-theoretic quantity, as a merit function (Setlur & Stone, 2016).

### Decoding assignment problem

We propose that when people interpret color-coding systems, they solve a decoding assignment problem. To do so, they make inferences about how the designer had mapped colors onto concepts while generating the color-coding system. In the decoding assignment problem in Fig. 2b, there are six colors and six objects that have been selected by the designer in the encoding assignment problem (Fig. 2a). The observer’s task is to infer the encoded assignments (dashed black lines), but how might they go about doing so?

### Color Inference Framework

The Color Inference Framework (Schloss, in press) proposes that people make inferences about colors (*color inferences*) based on an internal representation of color-concept associations that is stored in their minds. There are different kinds of color inference processes that operate on the same internal representation, which are modulated by perceptual context (e.g. colors in a color-coding system) and conceptual context (e.g. concepts in a color-coding system). Here, we aim to understand the assignment inference process for interpreting mappings between colors and concepts, which we believe enables people to solve decoding assignment problems.

We studied assignment color inference in the domain of recycling, where the color-coding system mapped different colored bins to different kinds of objects to be discarded. As described below, our approach was to manipulate input into the color inference system (i.e. the colors people saw in the experiments), measure the output of the system (i.e. people’s interpretations of how colors mapped onto objects to be discarded in our recycling task), and use those measures to evaluate hypotheses about how people make assignment color inferences.

#### Input for assignment inference

**Fig. 3:**
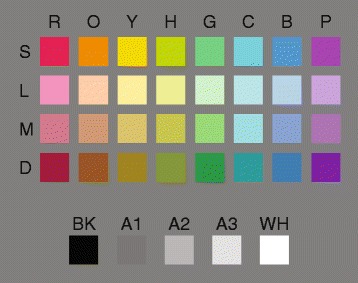
The BCP-37 colors: eight hues (*left* to *right*: Red, Orange, Yellow, cHartreuse, Green, Cyan, Blue, and Purple) sampled at four saturation/lightness levels (*top* to *bottom*: Saturated, Light, Muted, and Dark), plus five achromatic colors (*bottom row* from *left* to *right*: black (BK), dark gray (A1), medium gray (A2), light gray (A3), and white (WH). *Colors* in the figure are for illustration only and are not colorimetrically accurate. See Additional file 1: Table S1 for coordinates in CIE 1931 xyY space

**Fig. 4:**
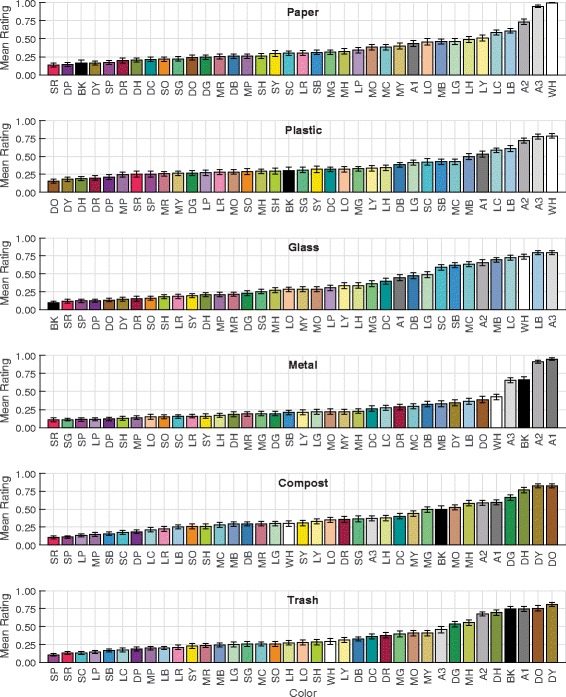
Mean color-object association ratings for paper, plastic, glass, metal, compost, and trash. Colors are sorted along the *x-axis* from most weakly associated to most strongly associated with each object. *Bar colors* represent the colors that were judged (also see x-axis label and corresponding coordinates in Additional file 1: Table S1). *Error bars* represent the ± standard errors of the means. See Additional file 1 for details on how the data were collected

We selected the colors for each experiment based on color-object association ratings obtained from 49 participants in a pilot experiment (see Additional file 1 for methodological details). In short, participants rated how strongly they associated each of the Berkeley Color Project 37 (BCP-37) colors (Palmer & Schloss, 2010; Schloss, Strauss, & Palmer, 2013) with each of six objects related to recycling: paper, plastic, glass, metal, compost, and trash. Approximations of the colors are shown in Fig. 3 and the CIE 1931 xyY coordinates are in Additional file 1: Table S1). The mean association ratings are displayed in Fig. 4, with the colors sorted from least associated to most associated with each object. We describe the details of how we selected the colors for Experiment 1 and Experiment 2 within the sections on each experiment below.

#### Output for assignment inference

To assess participants’ interpretations of how colors mapped onto objects to be discarded, we devised a recycling classification task. Participants saw images of unlabeled colored bins along with the name of object to discard (e.g. paper or trash) and they reported which colored bin was the correct one for discarding the object. It has previously been established that asking participants to interpret messages encoded in unlabeled perceptual features reveals how observers extract meaning from visual media (Zacks & Tversky, 1999).

#### Hypotheses about assignment color inference

We proposed and evaluated two hypotheses about how people perform assignment color inference. When people are given a single object and are asked to map it onto one color from a given set of colors, the *local assignment hypothesis* predicts that they simply match the object with its most strongly associated color. This means that two different objects could be mapped to the same color if that color is the strongest associate for both objects. In contrast, the *global assignment hypothesis* predicts that people not only consider the association strength between the object and candidate colors, but also account for the association strengths between all other objects and colors within the scope of the color-coding system. This can result in pairing colors with objects that are weakly associated if it results in better overall pairings for all objects considered.

In this study, we investigated assignment color inference in two experiments. Experiment 1 tested whether people perform local or global assignment in a simple scenario with two objects (paper and trash) and sets of two colors. We chose paper and trash because the colors most associated with these objects are distinct (see Fig. 4 and Additional file 1: Table S2). This avoids conflicts that arise from one-to-many and many-to-one mappings and therefore makes the task relatively easy, at least when one of the colors is strongly associated with paper and the other is strongly associated with trash. Experiment 2 tested whether people can still perform assignment inference with a larger set of six objects and six colors that contain conflicts due to one-to-many and many-to-one mappings. These conflicts make the task of selecting which six colors to use a nontrivial one. We determined which colors to use by designing different merit functions and solving the corresponding encoding assignment problems.

## Experiment 1

In Experiment 1, we compared the local assignment and global assignment hypothesis predictions for a simple case of discarding one of two kinds of objects into one of two unlabeled colored bins. Participants saw images of colored bins along with a description of a target object (paper or trash) and indicated which bin was appropriate for discarding the target object. We only tested paper and trash in this experiment because among all pairs of objects, this pair has the most different pattern of color-object association ratings, determined by the pilot experiment described above (also see Additional file 1: Table S2). We tested all pairs of four colors: the two colors that were most strongly associated with paper (white; WH) and trash (dark-yellow; DY) and two colors that were most weakly associated with paper (saturated-red; SR) and trash (saturated-purple; SP), see Fig. 4. Analyses verified that the “strong” color-object associations were stronger than the “weak” ones (see Additional file 1: Table S3).

**Fig. 5:**
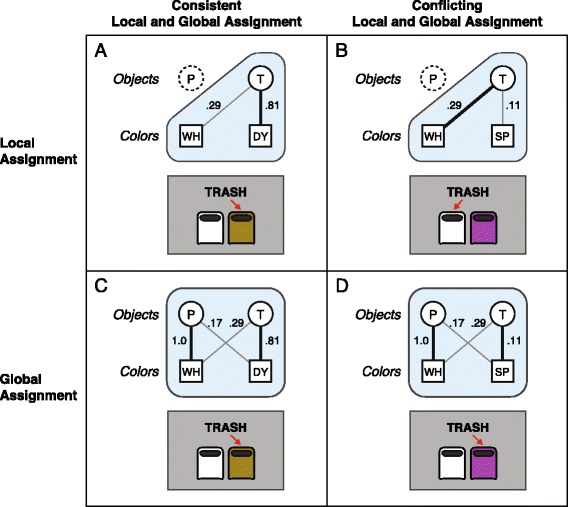
Illustration of the assignment inference process for two objects and two colors, under the local assignment (**a** and **b**) or global assignment (**c** and **d**) hypotheses. *Thick black lines* represent assigned color-object pairings and *thin gray lines* represent unassigned color-object pairings. Under local assignment, the target object (trash) is matched with its strongest associate (association ratings indicated on the graph edges). This means assigning trash to DY in the WH/DY color set (**a**) and assigning trash to WH in the WH/SP color set. Under global assignment, the target is matched by accounting for the association strengths of both objects and both colors. This means assigning trash to DY in the WH/DY color set (**c**) as in (**a**), but assigning trash to SP in the WH/SP color set (**d**), even though trash has a higher association rating with WH than with SP

Figure 5 illustrates the local and global assignment hypotheses for two color sets. Each panel contains two parts. The top part contains a bipartite graph showing the predicted assignments (thick black lines) between two possible objects (paper (P) and trash (T); circles) and two possible colored bins (squares). The bottom part shows the corresponding example trial, with a red arrow showing the predicted response.

The blue region in Fig. 5 represents the scope of objects and colors that serve as input into the assignment problem within each hypothesis. For the local assignment hypothesis, only the target object for the particular trial (in this case, trash) is in the scope and the other, non-target object (in this case, paper) is outside the scope. As a result, the target object always gets discarded into the bin whose color is most strongly associated with it, regardless of the association strength between the colors and the non-target object. As shown in Fig. 5a, trash should go in the dark-yellow bin when the color options are white and dark-yellow, and as shown in Fig. 5b trash should go in the white bin when the options are white and saturated-purple.

For the global assignment hypothesis, both the target and non-target are within the scope of the assignment problem. As a result, the target object gets discarded into the bin that allows for maximization of the total association strength over all possible color-object pairings. In Fig. 5c, the solution is the same as in the local assignment hypothesis (Fig. 5a), with trash going in the dark-yellow bin. However, in Fig. 5d, the solution conflicts with that of the local assignment hypothesis (Fig. 5b). Trash gets discarded in the saturated-purple bin even though trash is more strongly associated with white than with saturated-purple, because the global assignment specifies that white should be reserved for paper.

In this experiment, we quantified the predictions of the local and global assignment hypotheses and evaluated which predictions better capture participant responses.

## Methods

### Participants

There were 24 participants (mean age = 18.8, 17 women). All had normal color vision as screened using the H.R.R. Pseudoisochromatic Plates (Hardy, Rand, Rittler, Neitz, & Bailey, 2002), and all gave informed consent.^3^ The University of Wisconsin–Madison IRB approved the experimental protocol.

### Design and displays

As shown in Fig. 5, the displays contained images of two colored bins, one on the left and one on the right of the screen. The bins were 3.25 cm wide × 4.25 cm tall and were viewed from a distance of approximately 60 cm. Participants saw all six pairwise combinations of four colored bins (referred to as “color sets”). The colors were those that were most strongly associated with paper (white; WH) and trash (dark-yellow; DY), and the colors that were most weakly associated with paper (saturated-red; SR) and trash (saturated-purple; SP). The CIE 1931 xyY coordinates of the colors are listed in Additional file 1: Table S1. The colored bins were left/right balanced across trials. There was text at the top of the screen that indicated which target object was to be discarded on each trial (paper or trash). The full design included 6 color sets × 2 left/right color-bin assignments × 2 target objects × 6 replications, resulting in 144 trials.

The displays were presented on a 24.1-in. ASUS ProArt PA249Q monitor (1920 × 1200 resolution). The background was a medium gray (CIE x = 0.312, y = 0.318, Y = 19.26) that approximated CIE Illuminant C. We characterized the monitor using a Photo Research PR-655 SpectraScan® spectroradiometer and used it to verify accurate presentation of the colors. The deviance between the measured colors and target colors in CIE xyY coordinates was < 0.01 for x and y, and < 1 cd/m^2^ for Y. The experiment was programmed using Presentation (www.neurobs.com).

### Procedure

Participants were asked to imagine they had paper and trash to throw away and they wanted to figure out where the objects should be discarded. On each trial, there was text at the top of the screen indicating which object to discard on that trial (paper or trash), with a pair of colored bins below (Fig. 5). Participants indicated whether the object should be discarded in the left or right bin by pressing the left or right arrow key. Before the test trials, there were five practice trials. If participants asked questions about which bin they should choose, they were told to follow their intuition. The trials were presented in a random order separated by a 500-ms inter-trial interval. The participants were given a break after each set of 20 trials.

## Results and discussion

We generated predictions for the local and global assignment hypotheses using the color-object associations data from the pilot experiment described in Additional file 1 and shown in Fig. 4. To solve an assignment problem under the local assignment hypothesis, we simply match each object with its highest rated color. Under the global assignment hypothesis, we consider both objects and both colors together and pick the pairings that yields the largest total association rating.

One approach for generating these predictions might be to solve assignment problems under each hypothesis by calculating merit scores using the mean color-object association ratings presented in Fig. 4. This approach would be problematic because solving an assignment problem is a deterministic and absolute procedure. This means that if the outcome (e.g. whether “Trash” gets assigned to dark-yellow or saturated-red) hinges on whether one merit score is greater than another, the result will be the same regardless of whether the difference between these merit scores is large or small. However, we want predictions that reflect the sensitivity of outcomes to small changes in the merit scores.

To produce predictions with this sensitivity property, we used a sampling approach. We describe the sampling procedure for generating predictions in detail in Additional file 1. Roughly, we added small random perturbations to the association ratings in Fig. 4, solved the assignment problem using the perturbed values, repeated a large number of times, and then averaged all of the outcomes. This procedure has the desired effect because when association ratings are very different, adding small perturbations has little influence on the outcome of the assignment problem. However, when two association ratings are similar, perturbing them will sometimes cause a reversal in which association rating is largest and yield a different solution to the assignment problem. Repeating many times produces a distribution of outcomes that reflects the magnitude of the differences between merit scores. This approach should approximate the uncertainty in human judgments when different objects have similar color-object associations.

**Fig. 6:**
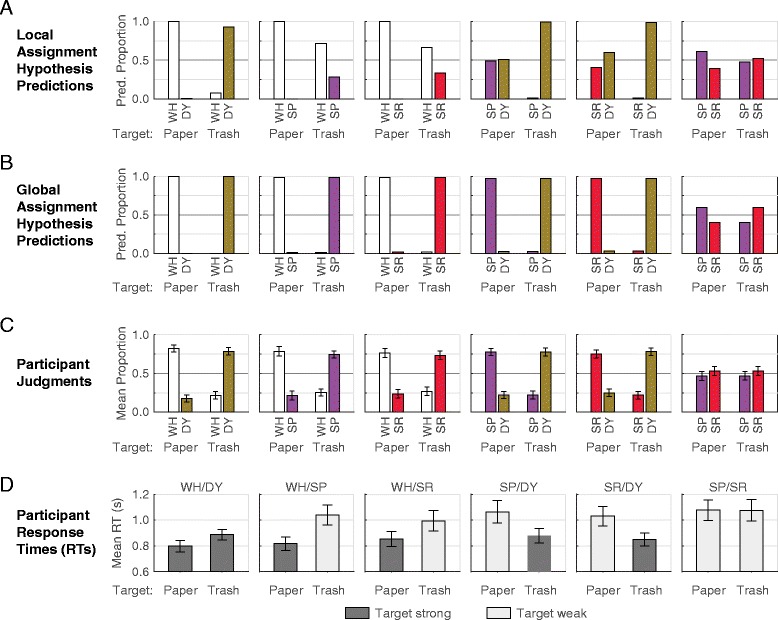
Predictions from (**a**) the local assignment hypothesis and (**b**) the global assignment hypothesis in terms of the proportion of times each color would be chosen for each target object (*x-axis*) within each color set (separate *plots*). The model predictions were generated from the color-object association data shown in Fig. 4. **c** The mean proportions of times participants chose each color for each target within each color set. **d** The corresponding mean response times (RTs) plot for each object within each color set. *Error bars* represent ± the standard errors of the means

Figure 6 shows the predictions of the local assignment hypothesis (Fig. 6a) and the global assignment hypothesis (Fig. 6b). It also shows the mean proportion of trials (out of 12) that participants chose each color within each color set for each object (Fig. 6c). The pattern of average responses across all objects and color sets was correlated with the predictions of the local assignment hypothesis (*r*(22) = 0.668, *p* < 0.001) and the global assignment hypothesis (*r*(22) = 0.991, *p* < 0.001), but the correlation with the global assignment hypothesis was significantly stronger (*z* = 6.13, *p* < 0.001). There are cases where the global and local assignment hypotheses make similar predictions, but where their predictions diverge, participants’ judgments appear to be more consistent with global assignment.

We also compared the two hypotheses at the individual participant level by conducting logistic regressions^4^ to predict each participant’s response on each trial using the predictions under each hypothesis and comparing the model fits across participants. We coded responses as 1 = left color chosen and 0 = right color chosen (12 trials in color set per object per participant).^5^ We coded predictors for the local and global assignment hypotheses as the probability of choosing the left color, using the values from Fig. 6a and b, respectively. For 18 out of 24 participants, the beta weights for the global assignment model were higher than the beta weights for the local assignment model. A sign test indicated that this distribution is significantly different from chance (*p* = 0.023), indicating that the global assignment hypothesis better predicted participant responses.

The results of this experiment demonstrate that people’s interpretations of the meanings of colors are highly context-dependent. They change from trial to trial, depending on the other colors in the scene. For example, participants almost always responded that red was correct for paper when paired with dark-yellow, almost never responded that red was correct for paper when paired with white, and responded near chance (0.5) when saturated-red was paired with saturated-purple (Fig. 6c).

Further, the results demonstrate that people can accurately interpret encoded assignments when none of the colors are strongly associated with the target object. They can do so as long as one of the colors is associated with the non-target object (at least when there are only two objects). For example, saturated-purple and dark-yellow are similarly weakly associated with paper (see Fig. 4 and Additional file 1: Table S2), yet participants systematically reported that saturated purple was the color for paper in SP/DY trials. They can do so because dark-yellow is strongly associated with trash (the non-target object for that trial), and solving the assignment problem tells them if dark-yellow is for trash, then saturated-purple must be the color for paper.

Although people can solve decoding assignment problems when both colors are weakly associated with the target, the response time (RT) data suggest doing so is more difficult compared to cases when one of the colors is strongly associated with the target (Fig. 6d). We analyzed the RT data by first calculating the median RT across all 12 trials within each condition. Figure 6d shows the mean of these median RTs across subjects for each condition, categorized as “Target strong” (one of the colors was strongly associated with the target) or “Target weak” (both colors were weakly associated with the target). RTs were significantly faster for the Target strong conditions (*t*(23) = 5.26, *p* < 0.001, *d*_*z*_ = 1.07), suggesting there is indeed a processing cost to solving the assignment problem when all of the colors are weakly associated with the target.

In summary, Experiment 1 provided evidence that participants approached interpreting the color-coding system in our task as a global assignment problem; they determined which assignments between colors and objects optimized the color-object associations of the entire set. This process sometimes resulted in observers interpreting that objects were intended to be assigned to colors that were their weakest associates, even when there was a stronger associate on the screen. However, there was a processing cost when the target did not have a strongly associated color in the color set.

## Experiment 2

In Experiment 1, we found evidence that people interpret color-coding systems by solving a decoding assignment problem with a global scope. Decoding was somewhat straightforward when there were only two objects and two colors. However, decoding becomes more complicated when there are several objects and colors and there are conflicts arising from one-to-many and many-to-one mappings. This is the case for the objects we studied in Experiment 2: paper, plastic, glass, metal, compost, and trash. As shown in Fig. 4 and Additional file 1: Table S2, color-object associations were very similar among paper, plastic, and glass, and among trash and compost.

Generally, a color-coding system should be easier to decode if: (1) each object has a color that is strongly associated with it; and (2) each object has only one color that is strongly associated with it. When these objectives are competing, we must choose how to prioritize one over the other. We tested two different color sets that were selected using two different merit functions: an *isolated merit function* and a *balanced merit function*. These merit functions trade off the relative weight placed on (1) and (2). We also tested a third set of colors that were selected using a *baseline merit function*, which attempts to select colors that are each equally associated with all the objects, thus maximizing confusion for the participants.

We tested the color sets above for two tasks. The “select task” asked participants to discard only one object into one of six bins on each trial (analogous to Experiment 1). The “match task” asked participants to simultaneously assign all six objects to six colors in a one-to-one manner. The match task can be viewed as a more sensitive measure of how well people decode the color-coding system because participants are explicitly asked to solve an assignment problem.

## Methods

### Participants

There were 96 participants divided into four groups (*n* = 24 per group, mean age = 19.0, 51 women), constructed from the orthogonal combination of two between-subjects factors (further detailed below): Task (select, match) × Optimized Color Set Group (isolated group, balanced group). All participants had normal color vision (screened using the H.R.R. Pseudoisochromatic Plates) and gave informed consent. The University of Wisconsin–Madison IRB approved the experimental protocol.

### Design, displays, and procedure

**Fig. 7:**
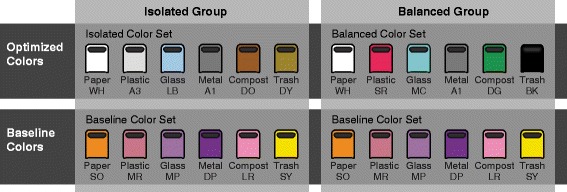
The color conditions in Experiment 2. Participants were divided into two Optimized Color Set Groups (Isolated Group and Balanced Group). Each group saw two color sets, an optimized color set (isolated or balanced) and the baseline color set

All participants saw two Color Set Types, an optimized color set and a baseline color set (Fig. 7). There were six bins within each color set, which corresponded to the six possible target objects: paper, plastic, glass, metal, compost, and trash. The bins were displayed in a row with six possible orders (left to right). The orders were defined by a Latin square design, ensuring that each bin color appeared equally often in each position, and all bin colors appeared equally often to the left/right of every other color. We did not test all possible orderings because of the combinatorial explosion.

Between subjects, there were two Optimized Color Set Groups, which determined the colors that were in the optimized color set (Fig. 7). Participants in the Isolated Group saw optimized colors that were selected using the isolated merit function and participants in the Balanced Group saw optimized colors that were selected using the balanced merit function. All participants saw the same baseline color set, selected using the baseline merit function. We varied optimized color sets between subjects because there were some overlapping colors between the two sets and we wanted to compare color sets generated using different merit functions without carryover effects across conditions.

We generated the colors for each color set by solving an encoding assignment problem as a linear program (Williams, 2013) using Matlab’s linprog function for each merit function. Using this approach, it takes < 30 ms to solve one assignment problem on a conventional laptop.

#### Isolated merit function

The isolated merit function is perhaps the most straightforward merit function for color-object assignments. It maximizes the color-object associations among all chosen color-object pairs. We call it the isolated merit function because it isolates the strongest association, ignoring the association strength between all unpaired colors and objects. So, if ${a}_{ij}$ is the mean color-object association rating of color $i$ with object $j$ (see Fig. 4), we would use

1 $$ {m}_{ij}={a}_{ij}. $$

**Fig. 8:**
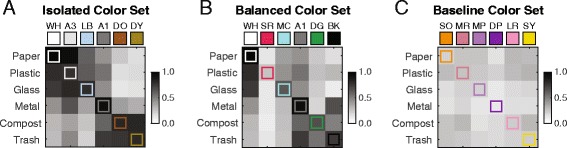
Color-object association matrices for all color-object pairs within each of the three color sets: (**a**) isolated, (**b**) balanced, and (**c**) baseline. Data are from the pilot experiment reported in Additional file 1 and are also shown in Fig. 4. The diagonal of each matrix indicates the encoded color-object assignment (correct answer) for each object

This function ensures that items are paired with their strongest associate, under the constraint that no color is assigned to more than one object. This can be observed in the color-object association matrix in Fig. 8a, where the assigned color-object pairs (diagonal of the matrix) have very strong color-object associations. However, using the isolated merit function can be problematic if some colors are strongly associated with multiple objects (one-to-many mappings) or multiple colors are associated with the same objects (many-to-one mappings). As shown in Fig. 8a, there are similar color-object associations among paper, plastic, and glass, and among compost and trash (strong associations off the diagonal of the matrix). This might lead to confusion in how to infer the correct assignments.

#### Balanced merit function

The balanced merit function mitigates conflicts due to one-to-many and many-to-one mappings by simultaneously maximizing the association between all paired items while minimizing the association between unpaired items. For example, if we assign color 9 to category 4, then we want ${a}_{94}$ to be large (correct assignment) and ${a}_{91},{a}_{92},{a}_{93},{a}_{95},{a}_{96}$ to be small (incorrect assignments). If, for instance, ${a}_{95}$ were also large, then the observers might be confused and not know whether object 4 or 5 is the best match for color 9. In defining the balanced association merit function, we used

2 $$ {m}_{ij}={a}_{ij}-\tau {\max}_{k\ne j}{a}_{ik}. $$

Here, the “max” is taken over all $k\in \left\{1,\dots, 6\right\}$ except for $k=j$. In other words, we subtract from each color-object association rating the largest color-object association rating among remaining pairings between that color and all other objects. The parameter $\tau \ge 0$ controls the degree to which we penalize strong associations between unpaired colors and objects. When $\tau =0$, we recover the isolated merit function ${m}_{ij}={a}_{ij}$. Increasing $\tau$ increasingly penalizes the biggest mismatch (the most confusing incorrect pair). The balanced merit function is defined as Eq. 2 when $\tau =1$.^6^ As shown in Fig. 8b, optimizing the balanced merit scores can lead to assigned color-object pairs that are not as strongly associated as they could be (weaker diagonal in the matrix), at the benefit of ensuring that associations between non-assigned color-object pairs is weak (weaker associations off the diagonal of the matrix).

#### Baseline merit function

As a baseline, we defined a merit function that we thought would cause people to respond at chance. There are many ways to achieve this, but the overarching criterion is that no object-color pair is a clear best choice; there are always competing choices. We used a merit function that ensures that for any object-color pairing, the association rating for the pair is comparable to the best association rating achievable by swapping the current object for a different one. Mathematically, the merit function is defined by:

3 $$ {m}_{ij}=-\mid {a}_{ij}-{\max}_{k\ne j}{a}_{ik}\mid . $$

As shown in Fig. 8c, the color-object association ratings are similarly low for all color-object pairings in the baseline set.

We did not use information-theoretic merit functions as were used by Lin et al. (2013) or Setlur and Stone (2016) because our input data (color-object association ratings; Fig. 4) are not probabilistic in nature. Although it might be possible to reweight our input data, interpret them as empirical probability distributions, and construct affinity scores similar to Lin et al. (2013) or Setlur and Stone (2016), we instead constructed deterministic metrics, which have similar qualitative properties but have a more natural interpretation in the context of our input data.

**Fig. 9:**
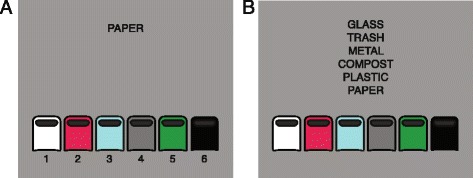
Example trials in the (**a**) select task and (**b**) match task in Experiment 2

In Experiment 2, we also tested two different tasks that varied between-subjects: select task and match task (Fig. 9). The monitor type and calibration procedure for both tasks were the same as in Experiment 1.

#### Select task

The select task was analogous to Experiment 1. On each trial, participants saw the name of one target object at the top of the screen and six bins in a row below (numbered 1 to 6) (Fig. 9a). They were asked to discard the object into one of the bins by pressing the corresponding number key at the top of the keyboard. Each participant completed 144 trials, resulting from the orthogonal combination of 2 color set types (optimized, baseline) × 6 objects to discard (paper, trash, glass, plastic, metal, compost) × 6 color bin orderings on the screen × 2 replications. Each trial was separated by a 500-ms inter-trial interval and participants received breaks after each set of 20 trials.

As in Experiment 1, participants could do this task by putting multiple objects in the same colored bins (no one-to-one constraint) and there was no requirement to account for the objects that were not queried on a particular trial when selecting a color. However, the global assignment hypothesis predicts that participants would take all objects into account, even though they were only judging one object at a time.

#### Match task

In the match task, participants were presented with all six objects simultaneously and were asked to match them with each of the six bins in a one-to-one manner (Fig. 9b). This task explicitly required participants to solve a decoding assignment problem. The displays in the match task were similar to the select task, except there were no numbers under the bins, and instead of text describing one object at the top of the screen, there was a list of all six objects (presented in a random order on every trial). Participants discarded each object into a bin by: (1) clicking on the object name to pick up the text; (2) moving the mouse to slide the object name onto a bin; and (3) clicking to drop the object in the bin. Once the object was dropped, its name appeared below the bin. Participants could change the object’s location after it had been dropped, by clicking on the name to return to its former position at the top of the screen, and then repeating the drag-and-drop procedure to place it in a different bin. Once participants were satisfied with their placement of all six objects, they pressed ENTER to go onto the next trial. Each participant completed 48 trials from the orthogonal combination of 2 color set types (optimized, baseline) × 6 color orderings × 2 replications. Within each trial, they matched each of six target objects, so the resulting dataset was analogous to the select task.

## Results and discussion

**Fig. 10:**
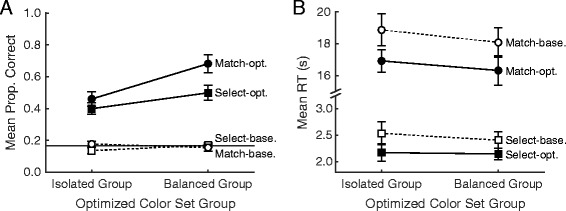
**a** Mean proportion of correct responses for the optimized color sets (*closed symbols*) and baseline color sets (*open symbols*) for participants in the isolated group and balanced group (*x-axis*), separated by participants in the match task (*circles*) and select task (*squares*). The horizontal line at 1/6 (approximately 0.17) represents chance. **b** Mean RTs for the optimized color sets (*closed symbols*) and baseline color sets (*open symbols*) for participants in the isolated group and balanced group (*x-axis*), separated by participants in the match task (*circles*) and select task (*squares*). Note the broken *y-axis* due to the responses for the select and match tasks being on different time scales. *Error bars* represent ± the standard errors of the means

Figure 10a shows the mean proportion of correct trials each Color Set Type (optimized, baseline) for participants in each Optimized Color Set Group (isolated, balanced) in each Task (select, match). Recall that each Optimized Color Set group saw different optimized color sets determined by the isolated and balanced merit functions but saw the same baseline color set (Fig. 7). The horizontal gray line represents chance (1/6; given six color options per trial). As expected, responses in the baseline condition did not significantly differ from chance (select task – isolated group: *t*(23) = –1.66, *p* = 0.111; select task – balanced group: *t*(23) = 0.24, *p* = 0.810; match task – isolated group: *t*(23) = 0.54, *p* = 0.592; match task – balanced group: *t*(23) = –0.55, *p* = 0.590).

We analyzed the accuracy data in Fig. 10a using mixed-effects logistic regression. Each trial was coded such that correct = 1 and incorrect = 0 (see Fig. 7 for specification of correct color-object pairings). The fixed effects were: Color Set Type (optimized vs baseline; within-subjects), Optimized Color Set Group (isolated vs balanced; between subjects), and Task (select vs match; between subjects), and their interactions. The random effects were random intercepts for subjects and random slopes for subjects for Color Set Type.

Overall, there were significant effects of Color Set Type (*β* = 1.99, *z* = 12.76, *p* < 0.001), Optimized Color Set Group (*β* = 0.52, *z* = 3.29, *p* < 0.001), and Task (*β* = 0.46, *z* = 2.88, *p* = 0.004). Accuracy was greater for the optimized color sets than the for baseline color set, for participants in the balanced group than in the isolate group, and for participants in the match task than in the select task. However, Color Set Type interacted with Optimized Color Set Group (*β* = 0.94, *z* = 3.02, *p* = 0.003) and with Task (*β* = 0.79, *z* = 2.54, *p* = 0.011), and there was a three-way interaction (*β* = 1.50, *z* = 2.44, *p* = 0.015).

To understand these interactions, we conducted a similar analysis, but separated the data for each task. As shown in Fig. 10a, responses were more accurate for the optimized color set than for the baseline color set within both the select task (*β* = 1.58, *z* = 8.55, *p* < 0.001) and match task (*β* = 2.46, *z* = 9.32, *p* < 0.001). Within the match task, Color Set Type interacted with Optimized Color Set Group (*β* = 1.79, *z* = 3.40, *p* < 0.001), revealing a benefit of optimizing using balanced merit function compared to the isolated function. There was no such interaction for the select task (*β* = 0.18, *z* = 0.50, *p* = 0.62), which suggests balanced optimization may not benefit performance when participants are only considering one object at a time.

Figure 10b shows mean RTs for each condition, regardless of whether responses were accurate. As in Experiment 1, we first calculated the median RT across the replications for each participant within each condition and conducted the analyses on those medians. We conducted separate analyses for the select and match tasks because they were not directly comparable—the select task involved discarding one object per trial, whereas the match task required assigning each of six objects to a unique color on each trial. Within each task, we conducted a 2 Color Set Types (optimized, baseline; within-subject) × 2 Optimized Color Set Group (isolated, balanced; between-subject) mixed-design ANOVA. Overall, RTs were faster for the optimized color sets than the baseline color sets within the select task (*F*(1,46) = 22.93, *p* < 0.001, ${\eta}_p^2$ = 0.33) and match task (*F*(1,46) = 8.23, *p* = 0.006, ${\eta}_p^2$ = 0.15). There was no effect of Optimized Color Set Group for (*F*s < 1) and no interaction (*F*s < 1) for either task. These results suggest there were greater processing costs for trying to interpret the encoded color-object assignments for the baseline color sets than for the optimized color sets, regardless of the merit function used to optimize the color sets or the task.

**Fig. 11:**
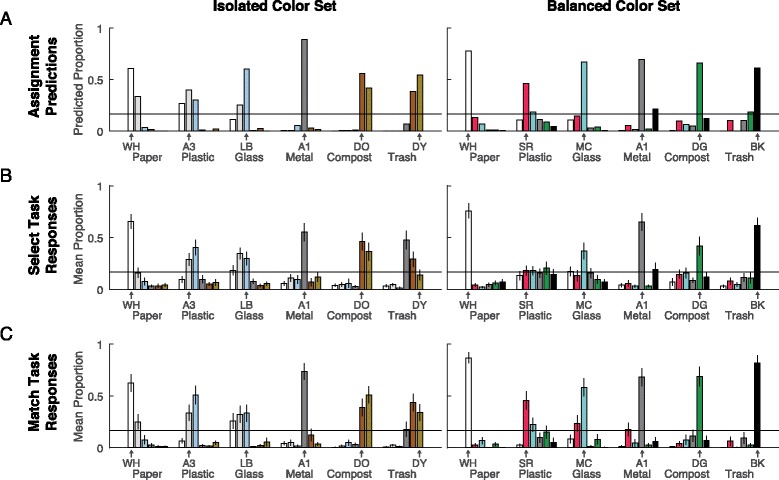
**a** Predictions of the proportion of times each color would be chosen for each object within the isolated color set and the balanced color set. **b** Mean proportion of times each color was chosen for each object within the isolated and balanced color sets for the select task and (**c**) for the match task. The correct response for each object is marked along the *x-axis* by an *arrow* with the color name pointing up at the correct color. *Error bars* represent the ± standard errors of the mean proportion across participants within each condition

Thus far we have focused on the responses averaged over objects. Now we examine the data separated by object. Figure 11 shows predicted responses and actual responses for each of the six objects for each optimized color set and task. We generated predictions based on the global assignment hypothesis for all six objects with each color set using the same procedure described in Experiment 1 and the Additional file 1.^7^ Figure 11a shows the predicted proportion of times each color should be chosen for each object, within the isolated and balanced color sets. Figure 11b and c show the mean proportion of times each color was chosen for each object within each color set, for the select and match tasks, respectively. The corresponding predictions and data for the baseline color set are in Additional file 1: Figure S3. As can be observed in Fig. 11, the model predictions were strongly correlated with the pattern of responses for both color sets within each task: select – isolated (*r*(34) = 0.80, *p* < 0.001), select – balanced (*r*(34) = 0.91, *p* < 0.001), match – isolated (*r*(34) = 0.91, *p* < 0.001), and match – balanced (*r*(34) = 0.96, *p* < 0.001). The strong correspondence between the predicted and actual responses suggests participants could successfully interpret the encoded color-object assignments.

We also compared the pattern of responses for the select task (Fig. 11b) and match task (Fig. 11c) within each color set and found they were strongly correlated (isolated color set: *r*(34) = 0.90, *p* < 0.001; balanced color set: *r*(34) = 0.93, *p* < 0.001). This suggests that participants generally approached the select task as though they had to find the best one-to-one correspondences between colors and objects (as participants in the match task had to do), even though participants in the select task could have put multiple object types in the same colored bin if desired. One notable exception is responses for plastic in the balanced color set (Fig. 11b). The encoding assignment problem assigned plastic to saturated-red, even though plastic is weakly associated with saturated-red, because it is more strongly associated with that color than any of the other objects are (Fig. 8b). The results suggest that people could not infer that plastic should be assigned to saturated-red due to this weak association if they judged one object at a time in the select task (Fig. 11b), but they could form that inference when they completed one-to-one assignments in the match task by a process of elimination (Fig. 11c). It is noteworthy that in the select task, participants did not appear to discard plastic in the white bin even though plastic is strongly associated with white (Fig. 8b), presumably because they were trying to solve an assignment problem with global scope and white is reserved for paper.

**Fig. 12:**
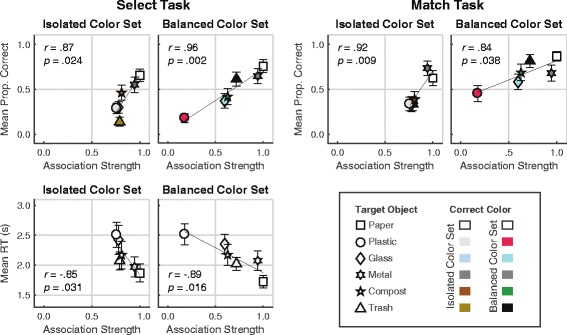
Mean proportion of correct responses as a function of color-object association strength (association ratings from Fig. 4) between objects and the correct color for each color set in the select and match tasks (*top row*). The color of the *points* represents the color of the correct response. Mean RTs as a function of color-object association strength between objects and the correct color for each color set in the select task (*bottom left*). There is no corresponding RT figure for the match task because there were not separate RTs for objects in the match task. The *points* are unfilled to indicate that these were mean RTs for all trials, regardless of whether responses were accurate. *Error bars* represent ± standard errors of the means

The next analysis aimed at understanding how the pattern of results related to color-object associations. As shown in Fig. 12, accuracy significantly increased as the color-object association strength increased. This was true for both the isolated and balanced color sets within the select and match tasks. Figure 12 also shows that RTs for each object decreased as the association strength between the object and the correct color increased in the select task. We could not test for a similar trend in the match task because there were no individual RTs for each object in the match task.

Although there is a positive correlation between association strength and accuracy for both color sets, this does not imply that one should maximize association strength when selecting colors (e.g. isolated merit function). As seen in Figs. 10a and 12, the balanced color set achieves a higher accuracy on average than the isolated color set even though it results in a larger spread of association strengths.

In summary, Experiment 2 showed that participants accurately decoded color-object assignments, even though there were extensive one-to-many and many-to one mappings. Participants were better at doing so when the assignments maximized association strength between assigned color-object pairings and minimized association strength between non-assigned color-object pairings, even though that required pairing objects with more weakly associated colors. However, responses were faster and more accurate when target objects were more strongly associated with their assigned color.

## General discussion

In this study, we investigated how people interpret color-coding systems used for visual communication. We approached this question by considering two types of assignment problems. There is an encoding assignment problem, in which a designer selects which color to pair with each concept in the color coding system, and a decoding assignment problem, in which the observer infers which color the designer paired with each concept in the color-coding system.

In Experiment 1, we used a recycling paradigm to test two competing hypotheses for how people approach the decoding assignment problem: the global assignment hypothesis and the local assignment hypothesis. Evidence supported the global assignment hypothesis. When deciding which colored bin to use for discarding paper or trash, people not only considered the association strength between the target object and candidate bin colors, but also accounted for the association strength between all other objects and colors within the scope of the color-coding system. This often resulted in discarding objects into colored bins that were weakly associated, if it resulted in better overall pairings for all objects considered.

In Experiment 2, we used a similar recycling paradigm with a larger set of objects and colors to test how people’s ability to decode color-coding systems is influenced by the merit functions used to generate the color sets in the encoding assignment problem. We tested two merit functions that produced two optimized color sets. The isolated merit function maximized the association strength between assigned color object parings without concern for associations between unassigned pairings. The balanced merit function simultaneously maximized the association strength between assigned color object parings while minimizing associations between unassigned pairings. Participants could reliably interpret both color sets, but they were more accurate for the balanced color set, primarily in the match task. These results suggest that it is easier to decode color-coding systems when they are designed to avoid strong associations between unassigned color-object pairs, even if that comes at a cost of reducing the overall color-object association strength for assigned color-object pairs.

We note that although we propose that people interpret color-coding systems by solving decoding assignment problems, we do not claim that there is some part of the brain that performs the same computations as in Matlab’s linprog function using the merit scores defined with Eq. 2. Instead, we argue that our results specify constraints on the underlying mechanisms that the brain implements when determining how colors correspond to objects in color-coding systems.

We also note that there is a conceptual distinction between color-object associations, such as rating how strongly white is associated with paper, and color inferences, such as inferring which colored bin is designated for paper. The color-object associations we present here are direct correspondences between colors and objects, given that participants in the pilot study were asked to rate how much they associated each color with each object. At the outset of this study, it was unknown whether participants would approach the recycling tasks by making inferences based on these color-object associations or if they would use some other approach. For example, although paper is strongly associated with white, that does not mean that people would infer that white was the correct bin for discarding paper. Participants could have associated white with the concept of *paper*, yet green with the concept of *recycling paper*, in which case they would have inferred the green bin was correct for discarding paper. Yet, we found that people inferred that white bins were for paper and green bins were for compost (Fig. 11), supporting the notion that assignment inference operates on color-object associations.

The present study focused on color-coding in recycling, but the results should generalize to any domain in which there are systematic associations between concepts and the colors that serve as their referents. The limitation, therefore, should depend on the degree to which there are systematic color-concept associations. Previous work on assigning colors to concepts for use in data visualization suggested such limitations might arise for concepts that are not highly “colorable” (e.g. types of fruit), where “colorabilty” was defined with respect to how systematically colors co-occurred with concepts in Google image searches (Lin et al., 2013). Subsequent work on color-concept assignments focused only on concepts that were highly colorable, where colorability was as defined with respect to co-occurrences between concepts and basic color terms (Berlin & Kay, 1969) in the Google n-grams corpus (Setlur & Stone, 2016). However, it is possible that a different method for quantifying color-concept associations—such as direct human judgments used here, or inferring color-word topics from human-designed media (Jahanian, Keshvari, Vishwanathan, & Allebach, 2017)—will reveal systematic associations useful for assigning more abstract concepts to colors for visual communication.

## Conclusion

The results of this study support the notion that people perform an assignment color inference process when they interpret color-coding systems. By understanding how people make such color inferences, it will be easier to anticipate observers’ expectations and create visual media that are easier for observers to interpret and understand.

## Additional file

Additional file 1: Supplementary methods, analyses, and results. (PDF 323 kb)

## Footnotes

1 By “designer,” we mean anyone who creates a visual display for communication purposes. This may be a professional designer, but it may also be a scientist who creates figures to communicate research findings, a hospital staff member who creates a color-coding system for organizing patient records, or a middle school student who creates a diagram for a class project. In the present study, the designers are the experimenters.

2 In general, it is possible that two different assignments achieve optimal total merit. For example, if all edges are assigned identical merit scores then any assignment is optimal. There are many efficient algorithms for solving assignment problems. The oldest and most famous method is the Kuhn–Munkres algorithm (Kuhn, 1955; Munkres, 1957).

3 One additional participant was run using the wrong (uncalibrated) computer monitor due to experimenter error, so that participant’s data were not analyzed or reported here.

4 This and all subsequent logistic regressions used generalized linear model (*glmer*) with a logit link function using the lme4 package (version 1.1-12) in R.

5 One participant on one trial pressed the enter key instead of the left or right arrow key so we flipped a coin to code their response on that trial.

6 In principle, one could choose any value of $\uptau$ to generate color sets, and this would control how much weight we place on maximizing the association between all paired items vs minimizing the association between unpaired items. The values $\uptau =0$ (no weight on minimizing the association between unpaired items) and $\uptau =1$ (equal weight on maximizing the association between all paired items and on minimizing the association between unpaired items) are representative choices, but other choices are possible. Additional file 1: Figure S1 shows the color sets that arise from all possible choices of $\uptau$. Additional file 1: Figure S2 shows results from data collected from a different set of participants on an intermediate value of $\uptau$ ($\uptau$ = 0.7). The pattern of the data for $\uptau$ = 0.7 lies between that of $\uptau$ = 0 and $\uptau$ = 1.

7 Originally, each of the three color sets was determined by solving an encoding assignment problem using a different merit function for each set (isolated merit function, balanced merit function, and baseline merit function). Here, we are trying to predict how participants solve the decoding assignment problem to recover the correct mapping between colors and objects. To generate these predictions, we tried both the isolated and balanced merit functions and both produced virtually identical predictions for the isolated color set (*r*(34) = 0.9991, *p* < 0.001) and the balanced color set (*r*(34) = 0.9996, *p* < 0.001). The predictions shown in Fig. 11a are for the balanced merit function.
