# Supplementary material for: Color inference in visual communication: the meaning of colors in recycling
Source: Cogn Res Princ Implic. 2018 Feb 21;3:5. doi: 10.1186/s41235-018-0090-y (PMC5820393; doi:10.1186/s41235-018-0090-y)
Supplement: Supplementary file 1 — Supplementary methods, analyses, and results. (PDF 323 kb) [file 41235_2018_90_MOESM1_ESM.pdf]

**Supplementary Material for:**

# Color inferences in visual communication: Interpreting the meanings of colors in recycling

Karen B. Schloss[1,2], Laurent Lessard[2,3], Charlotte S. Walmsley[4], Kathleen Foley[1,2]

[1]Department of Psychology, University of Wisconsin–Madison
[2]Wisconsin Institute for Discovery, University of Wisconsin–Madison
[3]Department of Electrical and Computer Engineering, University of Wisconsin–Madison
[4]Termeer Center for Targeted Therapies, Massachusetts General Hospital Cancer Center

## Pilot experiment: Color-object association ratings

This pilot experiment was conducted to obtain the color-object associations used in the merit scores for Experiment 1 and 2. Participants rated the association strength each of the BCP-37 colors and each of six objects related to recycling: paper, plastic, glass, metal, compost, and trash.

### Methods

**Participants.** There were 49 participants (mean age = 19.6, 33 females). All had normal color vision, screened using the HRR Pseudoisochromatic Plates (Hardy, Rand, Rittler, Neitz, & Bailey, 2002). All gave informed consent, and the Brown University IRB approved the experimental protocol.

**Design and Displays.** The colors were the BCP-37 colors (Figure 3), which included eight hues (red, orange, yellow, chartreuse, green, cyan, blue, and purple), sampled at four saturation/lightness levels (saturated, light, muted, and dark), in addition to 5 achromatic colors (white, light gray, medium grey, dark gray, and black). See Table S1 for CIE 1931 xyY coordinates and see Palmer and Schloss (2010) and Schloss et al., (2013) for details on how the colors were selected. The background was a medium gray (CIE $x$ = .312, $y$ = .318, $Y$ = 19.26) that approximated CIE Illuminant C. We characterized the monitor using a Minolta CS200 Chroma Meter and used it to verify accurate presentation of the colors. The deviance between the measured colors and target colors in CIE xyY coordinates was < .01 for x and y, and < 2 cd/m$^2$ for Y.

Each color was presented as a small square (100×100 pixels) at the center of the screen of a 24.1-in. ASUS ProArt PA246Q monitor (1920×1200 resolution). Below the square there was a 400-pixel long line-mark rating scale with the left endpoint labeled as "not at all," the right endpoint labeled as "very much," and a tick mark to denote the center of the scale. Responses were scaled to range from 0 to 1. There was black text at the top of the screen that named the object to be judged on each trial. Participants judged the association between each for the 37 colors with each of the 6 objects, resulting in 222 trials.

**Table S1.** Berkeley Color Project 32 (BCP-32) color names and corresponding CIE xyY coordinates (adapted from Schloss, Strauss, and Palmer, 2013).

| Color Name | | Code | x | y | Y |
|---|---|---|---|---|---|
| Red | Saturated | SR | 0.549 | 0.313 | 22.93 |
| | Light | LR | 0.407 | 0.326 | 49.95 |
| | Muted | MR | 0.441 | 0.324 | 22.93 |
| | Dark | DR | 0.506 | 0.311 | 7.60 |
| Orange | Saturated | SO | 0.513 | 0.412 | 49.95 |
| | Light | LO | 0.399 | 0.366 | 68.56 |
| | Muted | MO | 0.423 | 0.375 | 34.86 |
| | Dark | DO | 0.481 | 0.388 | 10.76 |

| | | | | | |
|---|---|---|---|---|---|
| Yellow | Saturated | SY | 0.446 | 0.472 | 91.25 |
| | Light | LY | 0.391 | 0.413 | 91.25 |
| | Muted | MY | 0.407 | 0.426 | 49.95 |
| | Dark | DY | 0.437 | 0.450 | 18.43 |
| Chartreuse | Saturated | SH | 0.387 | 0.504 | 68.56 |
| | Light | LH | 0.357 | 0.420 | 79.90 |
| | Muted | MH | 0.360 | 0.436 | 42.40 |
| | Dark | DH | 0.369 | 0.473 | 18.43 |
| Green | Saturated | SG | 0.254 | 0.449 | 42.40 |
| | Light | LG | 0.288 | 0.381 | 63.90 |
| | Muted | MG | 0.281 | 0.392 | 34.86 |
| | Dark | DG | 0.261 | 0.419 | 12.34 |
| Cyan | Saturated | SC | 0.226 | 0.335 | 49.95 |
| | Light | LC | 0.267 | 0.330 | 68.56 |
| | Muted | MC | 0.254 | 0.328 | 34.86 |
| | Dark | DC | 0.233 | 0.324 | 13.92 |
| Blue | Saturated | SB | 0.200 | 0.230 | 34.86 |
| | Light | LB | 0.255 | 0.278 | 59.25 |
| | Muted | MB | 0.241 | 0.265 | 28.90 |
| | Dark | DB | 0.212 | 0.236 | 10.76 |
| Purple | Saturated | SP | 0.272 | 0.156 | 18.43 |
| | Light | LP | 0.290 | 0.242 | 49.95 |
| | Muted | MP | 0.287 | 0.222 | 22.93 |
| | Dark | DP | 0.280 | 0.181 | 7.60 |
| Achromatic | Black | BK | 0.310 | 0.316 | 0.30 |
| | Dark Gray | A1 | 0.310 | 0.316 | 12.34 |
| | Med Gray | A2 | 0.310 | 0.316 | 31.88 |
| | Light Gray | A3 | 0.310 | 0.316 | 63.90 |
| | White | WH | 0.310 | 0.316 | 116.00 |

**Procedure.** Participants rated how much they associated each color with each object (paper, plastic, glass, metal, compost, and trash) on a continuous scale from "not at all" to "very much" by sliding a cursor along the scale and clicking to record their response. Trials were blocked by object, such that each color was rated for a given object before going onto the next object. The order of objects and colors within each object were both randomized. Trials were separated by a 500 ms inter-trial interval. The participants observed the monitor from approximately 60 cm away in a dark room. Before beginning the experiment, the participants anchored the endpoints of the scale (Palmer, Schloss, & Sammartino, 2013) by viewing a display of the full set colors and pointing to the colors that they most/least associated with each object.

**Results and Discussion**

Figure 4 in the main text shows the mean color-object association ratings for each object, sorted from low (left) to high (right) within each entity. There are similar patterns of color-object associations across multiple objects, especially between paper, glass, and plastic and between trash and compost. We quantified these relations by computing all 15 correlations between each pair of six entities (Table S2), using the Bonferroni correction to account for multiple comparisons (adjusted critical $\alpha = .003$).

**Table S2.** Correlations between mean color-object association ratings for all 15 pairs of 6 objects (see Figure 4 for color-object association ratings). Bolded *r* values indicate significant correlations after applying the Bonferroni correction for multiple comparisons (adjusted critical $\alpha = .003$).

| | Paper | Plastic | Glass | Metal | Compost | Trash |
|---|---|---|---|---|---|---|
| Paper | | | | | | |
| Plastic | **0.89*** ** | | | | | |
| Glass | **0.78*** ** | **0.89*** ** | | | | |
| Metal | **0.48** ** | **0.60*** ** | **0.48** ** | | | |
| Compost | -0.04 | -0.15 | -0.18 | **0.41*** | | |
| Trash | -0.01 | -0.05 | -0.16 | **0.64*** ** | **0.93*** ** | |

**p* < .05, ***p* < .01, ****p* < .001

**Comparisons of the color-object association ratings for the "strong" and "weak" colors in Experiment 1**

We verified that the "strong" color-object associations were stronger than the "weak" ones by conducting *t*-tests between all six pairs of colors for each object (Table S3). We applied the Bonferroni correction to account for 12 comparisons (adjusted $\alpha = .004$). The strong color for paper (WH) was more strongly associated with paper than any of the other three colors, and the strong color for trash (DY) was more associated with trash than with any of the other colors. The weak colors were equally associated with paper and equally associated with trash.

**Table S3.** *t* scores resulting of *t*-tests comparing the color-paper associations between each pair of the four colors and comparing the color-trash associations for each pair of the four colors. Bolded *t* values indicate significant *t*-tests after applying the Bonferroni correction for multiple comparisons (adjusted critical $\alpha$ = .004).

| | Paper | | | Trash | | |
|---|---|---|---|---|---|---|
| | WH | DY | SR | WH | DY | SR |
| DY | **29.52*** ** | | | **10.27*** ** | | |
| SR | **32.92*** ** | 1.03 | | **3.75*** ** | **19.44*** ** | |
| SP | **29.56*** ** | 0.04 | 1.12 | **4.67*** ** | **22.76*** ** | 0.97 |

****p* < .001

**Generating predictions for the local and global hypotheses**

Given association ratings, we can predict participants' responses in the recycling task by matching each object with its highest-rated color (local assignment hypothesis) or by considering all objects and colors within the scope (global assignment hypothesis). The predictions generated in this way are the result of a deterministic procedure so they always produce the same predictions when given the same association ratings. Using a deterministic procedure is problematic because it does not capture the sensitivity of the result to small changes in the association data. A prediction might hinge critically on whether one association rating is larger than another or vice versa. If the two ratings are very similar, we would like the prediction to return an intermediate value.

For example, suppose the mean associations between two objects and two colors (on a scale from 0 to 1) are as shown below:

| | **Color 1** | **Color 2** |
|---|---|---|
| **Object 1** | .53 | .47 |
| **Object 2** | .48 | .51 |

Solving an assignment problem using these mean associations as the merit scores would match Color 1 to Object 1 and Color 2 to Object 2 100% of the time. However, these associations were obtained by averaging the ratings of many individuals, so a particular individual's association rating could be different enough to cause them to match Color 1 to Object 2 and Color 2 to Object 1 instead. We would expect that if the color-object associations are statistically equivalent, then participants would respond at chance when deciding how colors should be assigned to objects in our recycling task. Therefore, we used a procedure that incorporates variability in the color-object association ratings across participants to generate more sensitive predictions than if we had just solved the assignment problem once on the mean color-object association data.

We adopted a simulation-based method to form our predictions. Recall that the association ratings from the pilot experiment (Figure 4) are averaged over 49 participants. Each time a participant rated a color, they used a continuous scale which led to digitized ratings from $-100$ to $+100$. To provide results that capture uncertainty in these data, we assumed each of the ratings was subject to additive uncertainty (normally distributed, zero-mean with a standard deviation of 10, and identically and independently applied to all participants and all ratings). We then performed the following averaging procedure:

1. For each of the participants in the pilot experiment:
   a. Perturb the subject's ratings using normally distributed noise as described above.
   b. For each realization of the ratings, solve the local/global assignment problem. This results in a sequence of absolute predictions (0's and 1's) for each matching task.
   c. Repeat the above 100 times using a different random noise realization each time.
2. Repeat the above for each of the 49 subjects in the pilot experiment.
3. This results in 4,900 absolute predictions. Compute the average over all predictions and return this as the final prediction from the model.

For example, in the case where "Trash" must be discarded into either a red (SR) or purple (SP) bin, the result might hinge on whether the association rating between Trash and red is larger or smaller than the rating between Trash and purple. By randomly perturbing all the ratings, solving the problem for each instance, and averaging the results, we obtain a prediction that is not absolute (e.g. pick red some proportion of the time and purple the rest of the time) that reflects both variations across the individuals in the pilot study as well as uncertainty in the mechanism by which ratings were estimated for each individual. In cases where one rating is much larger than another, such as Paper – white being much stronger than Paper – red, perturbing the data has little to no effect on the predictions.

## Variations in $\tau$ for creating optimized color sets

In Experiment 2 in the main article, we focused on two values of $\tau$ when calculating the optimized color sets (Equation 2): $\tau = 0$, which is the isolated color set and $\tau = 1$, which is the balanced color set. Figure S1 illustrates those two color sets, along with all possible other color sets generated from $\tau = 0$ to $\tau = \infty$.

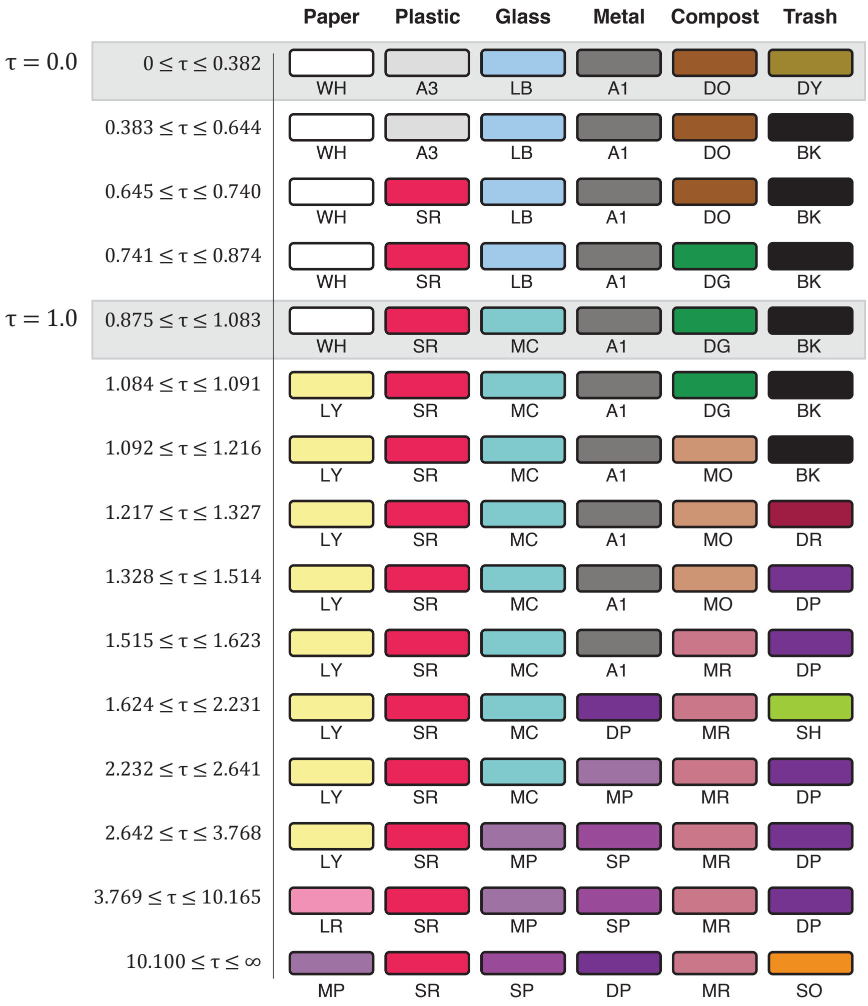


**Figure S1.** Color sets that arise from all possible choices of $\tau$. Values of $\tau$ are binned because the color sets are identical within those ranges of $\tau$ (rows).

In addition to testing $\tau = 0$ and $\tau = 1$ reported in the main article, we tested an independent group of participants in the select and the match tasks using the $\tau = .7$ color set. The methods were identical to Experiment 2. The data presented in Figure S2 are for 24 participants in the select task and 24 participants in the match task (mean age 19.4, 27 females). One additional participant was run in the select task but the data were excluded because the participant did not have typical trichromatic color vision (screened using the H.R.R. Pseudoisochromatic Plates). Comparing the pattern of results for the $\tau = .7$ color set to the isolated ($\tau = 0$) and balanced ($\tau = 1$) color sets (Figure 11), it appears the $\tau = .7$ results (Figure S2) were intermediates between the results of $\tau = 0$ and $\tau = 1$.

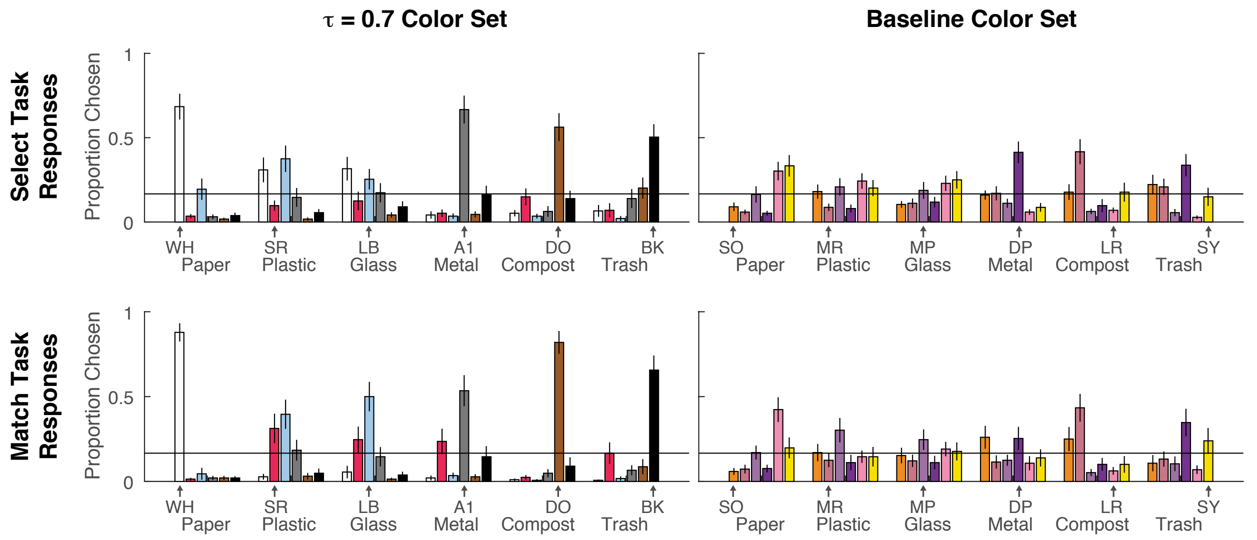


**Figure S2.** Mean proportion of times each color was chosen for each object, for the $\tau = .7$ and baseline color sets within each task type. The correct response for each object is marked along the x-axis with the color name and arrow pointing up at the correct bar. Error bars represent the +/- standard errors of the mean proportion across participants within each condition.

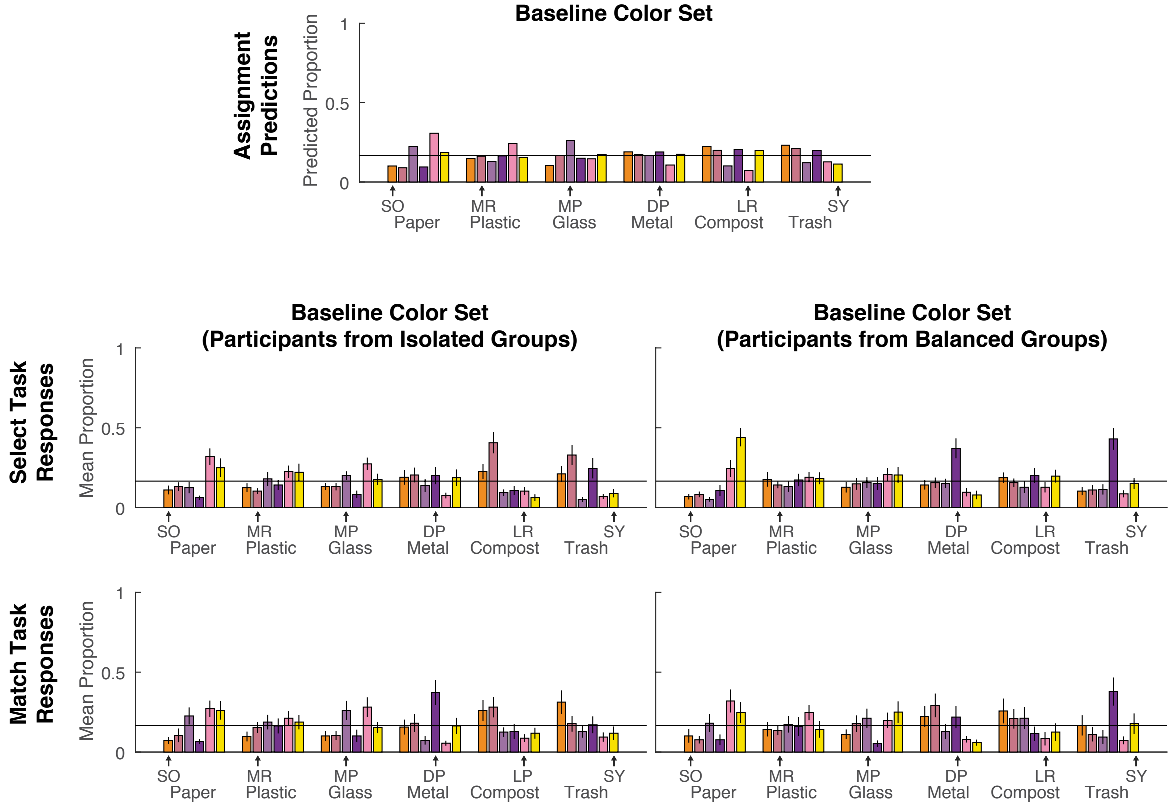


**Figure S3.** Assignment predictions (top) and mean proportion of times each color was chosen for each object for the baseline color set, corresponding to participants in the isolated color set group and the balanced color set group within each task type. The correct response for each object according to the baseline merit function is marked along the x-axis with the color name and arrow pointing at the correct bar. Error bars represent +/- standard errors of the mean proportion across participants within each condition.
